# Glucose Metabolism in Osteoblasts in Healthy and Pathophysiological Conditions

**DOI:** 10.3390/ijms22084120

**Published:** 2021-04-16

**Authors:** Antonia Donat, Paul-Richard Knapstein, Shan Jiang, Anke Baranowsky, Tobias-Malte Ballhause, Karl-Heinz Frosch, Johannes Keller

**Affiliations:** Department of Trauma and Orthopedic Surgery, University Medical Center Hamburg-Eppendorf, 20246 Hamburg, Germany; a.donat.ext@uke.de (A.D.); p.knapstein@uke.de (P.-R.K.); s.jiang.ext@uke.de (S.J.); a.baranowsky@uke.de (A.B.); t.ballhause@uke.de (T.-M.B.); k.frosch@uke.de (K.-H.F.)

**Keywords:** osteoblast, glucose, diabetes, metabolism, molecular signaling

## Abstract

Bone tissue in vertebrates is essential to performing movements, to protecting internal organs and to regulating calcium homeostasis. Moreover, bone has also been suggested to contribute to whole-body physiology as an endocrine organ, affecting male fertility; brain development and cognition; and glucose metabolism. A main determinant of bone quality is the constant remodeling carried out by osteoblasts and osteoclasts, a process consuming vast amounts of energy. In turn, clinical conditions associated with impaired glucose metabolism, including type I and type II diabetes and anorexia nervosa, are associated with impaired bone turnover. As osteoblasts are required for collagen synthesis and matrix mineralization, they represent one of the most important targets for pharmacological augmentation of bone mass. To fulfill their function, osteoblasts primarily utilize glucose through aerobic glycolysis, a process which is regulated by various molecular switches and generates adenosine triphosphate rapidly. In this regard, researchers have been investigating the complex processes of energy utilization in osteoblasts in recent years, not only to improve bone turnover in metabolic disease, but also to identify novel treatment options for primary bone diseases. This review focuses on the metabolism of glucose in osteoblasts in physiological and pathophysiological conditions.

## 1. Introduction

Understanding the interactions of bone and glucose metabolism has strongly been brought to the fore within the last years. As a matter of fact, both are associated closely and interact on multiple levels, and recent research has focused on the dynamic processes of energy and substrate utilization in osteoblasts. In turn, bone tissue was suggested to also act as an endocrine organ itself, releasing bioactive osteocalcin (OC) and thereby increasing pancreatic beta-cell proliferation as well as insulin secretion [1]. Furthermore, insulin levels correlate positively with bone remodeling markers, implicating a feed-forward mechanism between osteoblast activity and metabolic control [2,3]. Bone underlies ongoing transformation, and its quality mainly relies on the delicate balance between osteoblast and osteoclast activity. In this respect, osteoblast dysfunction has been found to be caused by calorie restriction in anorexia nervosa, and also by hyperglycemic states in diabetes [4,5]. Ongoing research and further investigation of the underlying molecular principles may provide the basis for improved treatment of diabetes-induced bone fragility. This review aims to summarize current knowledge on osteoblast and glucose metabolism, as well as the most recent experimental and clinical data available, focusing on osteoblasts as a main target for osteoanabolic drugs.

## 2. The Osteoblast in Bone Physiology

**Figure 1:**
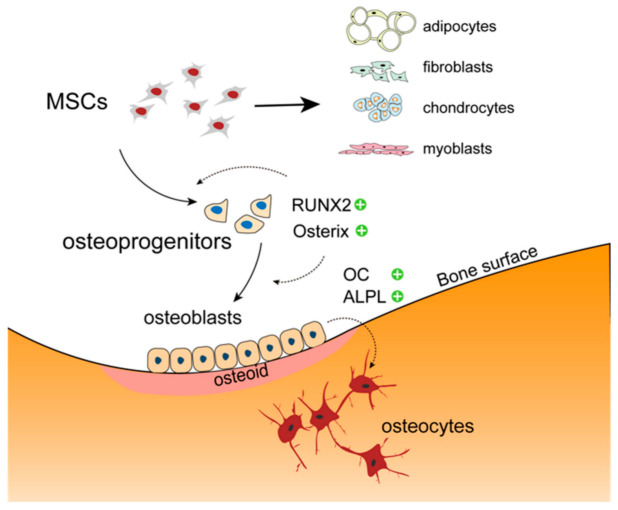
Osteoblasts are derived from mesenchymal stem cells (MSCs), precursor cells also giving rise to chondrocytes, adipocytes, myoblasts and fibroblasts. Osteoblasts aggregate along bone surfaces to synthesize osteoid. Runt-related transcription factor (RUNX2) is one of the key transcription factors required for osteoblastic differentiation and is highly expressed at both early and late stages of differentiation. Osteocalcin (OC) produced by osteoblasts is expressed during bone mineralization. Alkaline phosphatase (ALPL) represents the key enzyme for driving bone matrix mineral deposition.

Osteoblasts are cells of major importance in bone remodeling and glucose homeostasis. Together with bone-resorbing osteoclasts and bone matrix-embedded osteocytes, they represent the main cellular components of skeletal tissue. The leading functions of mature osteoblasts include the synthesis of collagen type I, the most abundant organic matrix component in bone ensuring biomechanical stability and tensile strength. Unlike osteoclasts, osteoblasts are derived from mesenchymal stem cells (MSCs), precursor cells also giving rise to chondrocytes, adipocytes, myoblasts and fibroblasts [6,7]. Under the influence of runt-related transcription factor (RUNX2) and osterix, MSCs differentiate into pre-osteoblasts and eventually into osteoblasts (Figure 1). 

RUNX2 is one of the key transcription factors required for osteoblastic differentiation. It is synonymously known as core binding factor alpha1 (CBFA1) and was first described in 1997 [8,9,10,11]. RUNX2 not only promotes the expression of genes encoding the main collagen component (alpha-1 type I collagen; *COL1A1*) and the osteoblast-specific protein osteocalcin (OC; *BGLAP*) in the osteoblast lineage, but is also essential for the transition into an osteoblastic phenotype when expressed in fibroblasts. In a murine model, homozygous deletion of RUNX2 leads to a complete lack of ossification, resulting in embryonic lethality [10]. As OC expression is barely measurable in those mutant mice, a disruption of early osteoblastic differentiation has been demonstrated [11]. Mice with a heterozygous deletion of RUNX2 display impaired intramembranous ossification but lack any other skeletal abnormalities [12]. Paradoxically, overexpression of RUNX2 leads to osteoblast dysfunction with diminished quality of cortical bone and indicates a repressive function on osteoblasts in late stages of differentiation [13]. Overall, good comparability between human and murine RUNX2 expression exists [14], so that employment of in vivo and murine primary osteoblasts in vitro represent adequate models for the investigation of molecular principles underlying human bone diseases.

Osterix, also known as transcription factor Sp7, drives intramembranous and endochondral ossification through enhanced collagen type 1 and osteocalcin expression [15,16]. It furthermore acts as a regulator of MSC fate, inhibiting chondrogenesis in favor of osteogenesis [17]. A mouse strain with postnatal global knockout of osterix displays short limbs, trabecular absence and fragile cortical bone accompanied by an accumulation of calcified cartilage [18]. An osteopenic bone phenotype was also found in postnatal conditional knockout of osterix [19], confirming the strong impact of osterix on osteoblastic differentiation. 

Osteoblastic alkaline phosphatase (ALPL) represents the key enzyme to drive mineral matrix mineralization. The membrane-bound protein is encoded by *ALPL*, which is also a direct target of RUNX2 [20]. ALPL hydrolyzes pyrophosphate into phosphate, which is then incorporated in the bone matrix at the mineral deposition site. In osteoblastic precursor cells, expression of ALPL correlates with the start of mineralization in a time-dependent manner [21]. Cultures of calvaria-derived osteoblasts from ALPL-deficient mice form organized nodules but fail to carry out the process of mineralization [22]. Overall, serum ALPL levels correlate with osteoblastic differentiation and activity and are used to support clinical diagnosis, as they are elevated in diseases with higher levels of bone remodeling, such as Paget’s disease of bone and hyperparathyroidism. Reduced levels of ALPL are observed not only in children with diabetes, but also in adults with diabetes-induced osteopenia [23,24]. In hypophosphatasia, a rare hereditary disease caused by different loss-of-function mutations of *ALPL* [25], inorganic pyrophosphate accumulates extracellulary. The histopathological bone phenotype is determined by a reduced mineral density and emulates the diseases rickets and osteomalacia, which are caused by a lack of vitamin D and/or calcium and are characterized by bone deformities, reduced mineralization rates and increased fracture risk [26,27]. Further clinical observations range from benign dental disorders to lethal forms, depending on the specific mutation and correlating with the age of onset [28,29]. Accordingly, idiopathically reduced serum ALPL activity is accompanied by reduced bone turnover in otherwise healthy subjects [30].

Several regulator mechanisms ensure a balanced state of bone resorption and the synthesis of new bone. For instance, osteoclasts secrete substances in a paracrine manner, thereby recruiting osteoblast precursors to the site of bone resorption and stimulating bone-forming osteoblasts [31,32]. Likewise, osteoblasts secrete receptor activator of nuclear factor kappa b ligand (RANKL), which binds to the receptor activator of nuclear factor kappa b (RANK) on osteoclast precursor cells and stimulates their differentiation into osteoclasts. Osteoprotegerin (OPG), another glycoprotein secreted by osteoblasts, acts as a soluble decoy receptor for RANKL, thereby inhibiting RANKL-induced osteoclast differentiation and bone resorption to ensure a well-balanced state of bone resorption and construction. Its expression is enhanced by RUNX2 in a direct manner [33]. In vitro experiments demonstrate that OPG is further capable of inducing apoptosis of osteoclasts, even though these effects have not been verified in vivo [34,35]. Once osteoblastic differentiation commences, the RANKL/OPG ratio shifts towards OPG, enabling bone formation without the counteracting effects of increased bone resorption [36]. After a life span of around 3 months, osteoblasts either undergo apoptosis or become embedded into the bone matrix, where they terminally differentiate into osteocytes without the ability to proliferate. Osteocytes regulate phosphate homeostasis via secretion of fibroblast growth factor 23 in an endocrine manner [37]. Furthermore, they adjust bone building activity to physical activity through synthesis of sclerostin, as recently reviewed by Uda et al. [38].

## 3. Interactions between Glucose Homeostasis and Osteoblasts

### 3.1. Osteoblastic Glucose Receptors and Glucose Utilization

From an energetic point of view, bone remodeling is a costly process, and osteoblasts primarily rely on glucose as their main carbon and energy source [39,40]. To facilitate glucose uptake, osteoblasts express three of the four known members of the family of glucose transporters (GLUT1-4), namely, GLUT1, GLUT3 and GLUT4. The energetic requirements of osteoblast differentiation are met by a fast-forward mechanism between glucose transporter GLUT1 and the osteoblast transcription factor RUNX2. In particular, higher levels of intracellular glucose inhibit proteasomal degradation of RUNX2. RUNX2 itself enhances GLUT1 expression by binding and activating the *Glut1* promotor in a direct manner, thereby facilitating glucose uptake and enhancing osteoblastic differentiation [41]. While the expression levels of GLUT1 and GLUT3 do not change significantly during all stages of osteoblast differentiation, GLUT4 expression may increase up to five fold in an insulin-dependent manner. In contrast, expression of GLUT2 is not detectable in osteoblasts [42]. A homozygous deletion of GLUT1 in mice leads to embryonic lethality, whereas mice with an osteoblast-specific deletion of GLUT1 exhibit reduced bone volume with low levels of OC, as well as impaired glucose tolerance [41,43]. A global GLUT4 knockout model results in growth retardation and several other abnormalities most pronounced in cardiac and adipose tissue [44]. In contrast, mice with osteoblast/osteocyte-specific deletion of GLUT4 display endocrine anomalies comparable with those observed in mice with osteoblast-specific GLUT1 inactivation. However, they do not exhibit an apparent bone phenotype, confirming a direct role of GLUT1 rather than GLUT4 in osteoblasts [42].

**Figure 2:**
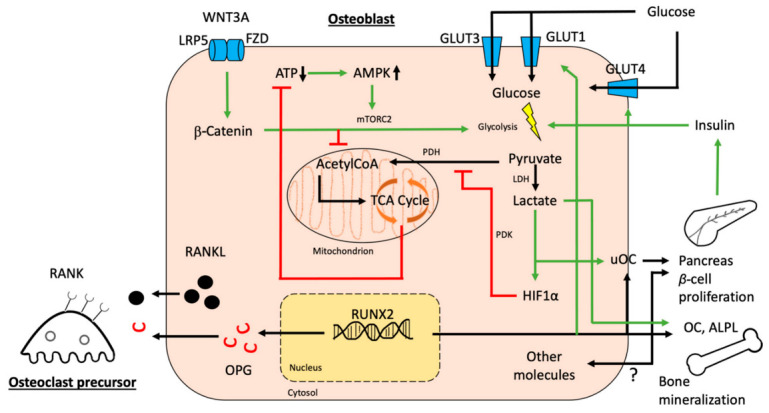
An overview of osteoblastic glucose pathways and secreted products. Intracellular energy metabolism relies on different feed-forward and negative feedback mechanisms. Abbreviations: LRP5 = low-density lipoprotein receptor-related protein 5; FZD = frizzled; GLUT1-4 = glucose transporters 1-4; ATP = adenosine triphosphate; AMPK = 5’ AMP-activated protein kinase; mTORC2 = mammalian target of rapamycin complex 2; TCA = tricarboxylic acid; (u)OC = (uncarboxylated) osteocalcin; HIF1α = hypoxia induced factor 1alpha; ALPL = alkaline phosphatase; RUNX2 = runt-related transcription factor; OPG = osteoprotegerin; RANKL = receptor activator of nuclear factor kappa b ligand; RANK = receptor activator of nuclear factor kappa b; PDK = pyruvate dehydrogenase kinase; PDH = pyruvate dehydrogenase; LDH = lactate dehydrogenase.

After passive diffusion along a concentration gradient through GLUT receptors from extracellular to intracellular, glucose molecules are subjected to aerobic glycolysis, and to a lesser extent, subsequently enter the tricarboxylic acid (TCA) cycle, even under aerobic conditions. In vitro studies and results from measuring metabolites of glycolysis and TCA cycle in mice via radiolabeled glucose show that osteoblasts primarily rely on energy generation through glycolysis rather than on the TCA cycle [45,46]. This unusual cellular glucose utilization, called the Warburg effect, has primarily been observed in cancer cells, where lactate is produced from glucose under replete oxygen conditions. Local oxygen tensions in bone depend on the specific location and range between 10 to 60 mmHg, with the lowest oxygen tension being observed in the bone marrow cavity [47]. In comparison, brain microvascular environment of mice displays mean pO_2_ concentrations of around 33 mmHg [48]. Oxygen levels in the bone marrow cavity might therefore be considered as rather hypoxic but still sufficient to generate ATP through completion of the TCA cycle [40]. During glycolysis, glucose is converted into pyruvate with a net profit of two molecules of adenosine triphosphate (ATP). To maintain glycolysis, electron donor nicotinamide adenine dinucleotide (NAD^+^) needs to be restored. Therefore, the end-product pyruvate is converted into lactate, which then stabilizes hypoxia induced factor 1alpha (HIF1α) and indirectly increases glycolysis using altered gene expression as well as a positive feedback loop through further induction of osteoblast differentiation via end-product lactate (Figure 2, [49]). A mouse model with a specific deletion of von-Hippel–Lindau protein in osteoblasts, resulting in the stabilization of HIF1α and thus hypoxic cell metabolism, displays a high bone mass phenotype with reduced blood glucose levels, explained by a massive induction of osteoblastic glycolysis [50]. Interestingly, this phenotype is independent of the enhanced blood vessel formation, but susceptible to glycolytic suppression. Regan et al. furthermore showed that HIF1α enters the nucleus to enhance the expression of multiple target genes, including pyruvate dehydrogenase kinase 1, which then phosphorylates and inactivates pyruvate dehydrogenase, so that the transition from glycolysis into the TCA cycle is blocked [51]. When cultured under hypoxia, mature osteoblasts display elevated intra cellular and extracellular lactate levels and are able to uncouple glycolysis from the TCA cycle, which is consistent with higher resistance and survival at low oxygen levels and the observations mentioned beforehand [52]. These findings clearly indicate an enhancing effect of hypoxia on glycolysis, even though therapeutic usage of the underlying molecular mechanisms has not been established yet.

In accordance with the research carried out by Regan et al. [51], murine conditional PDK1 knockout strains exhibit decreased bone mass and trabecular number [53,54]. Out of the four isoenzymes PDK1-4, PDK2 increases predominantly during osteoblastic differentiation but exacerbates the bone phenotype of an osteoporotic mouse model, as inhibition of PDK2 reduced RANKL-mediated osteoclast activation and improved bone quality [55]. Similar effects have been observed in a different mouse model, where PDK4 deletion restored bone loss secondary to immobility [56]. These inconsistent findings may derive from different functions of PDK isoenzymes in physiological and pathological states and should be elaborated in future research. 

The prominence of aerobic glycolysis in osteoblasts is indeed surprising, given the fact that this pathway yields only two ATP molecules per glucose molecule, while the complete oxidation of pyruvate through the TCA cycle yields 30–32 ATP molecules [57]. One possible explanation could be an inhibitory effect of excessive ATP levels on osteoblastic mineralization, which was observed at least in vitro [58]. Furthermore, aerobic glycolysis consumes glucose at a faster pace and yields multiple glycolytic intermediates, which can be used by osteoblasts through alternative pathways. In cancer cells, aerobic glycolysis has been suggested to enhance cell proliferation through fast ATP production and the generation of crucial metabolic intermediates required for active lipid and nucleotide synthesis [59,60]. Similar mechanisms may be relevant in osteoblasts, which do not show high proliferation rates in vivo, yet synthesize large amounts of matrix proteins requiring carbon atoms derived from glycolysis. Moreover, aerobic glycolysis was suggested to be associated with the active release of citrate, which is essential for the formation of apatite nanocrystals in bone [61,62]. 

As in many other cells, the main glycolytic enzymes in osteoblasts are induced during ATP depletion due to energy-consuming processes, such as cell differentiation and bone formation. In the course of these metabolically challenging states, adenosine monophosphate activated protein kinase (AMPK) is phosphorylated and highly active [63]. A unique characteristic of osteoblasts is the induction of glycolysis by the WNT ligand WNT3A, which promotes its actions through activation of mammalian target of rapamycin complex 2 (mTORC2). Radiolabeled tracing of glucose-derived CO_2_ showed that WNT3A inhibits glucose from entering the TCA cycle and reduces levels of acetyl-CoA in the nucleus through suppression of citrate levels. Consequently, histone acetylation is reduced and transcription factors such as RUNX2 can bind to the DNA [64,65]. As these results were published recently and have yet not been replicated by other investigators, they should be evaluated carefully. Paradoxically, WNT3A is also responsible for keeping an undifferentiated MSC population and suppressed osteogenic differentiation in vitro [66]. These varying observations lack a profound explanation, even though different metabolic phenotypes of the stromal ST2 cell line and multipotent human MSCs, which were used in the corresponding experiments, might have an influence on the variability of results.

Several other mechanisms also increase aerobic glycolysis in osteoblasts. During early osteoblast differentiation, insulin-like growth factor 1 (IGF1) enhances glycolysis through activation of AMPK, whereas downregulation of AMPK with concomitant activation of mTORC2 is observed in later stages of differentiation [67]. Similarly, transforming growth factor beta (TGFβ) binds to type I and II transmembrane receptors and increases intracellular β-catenin, which then translocates into the nucleus and has been proposed to increase the expression of glycolytic enzymes such as PDK1 and lactate dehydrogenase A [64,68,69]. While endogenous parathyroid hormone (PTH) stimulates bone resorption through increased expression of RANKL and degradation of RUNX2 [70], clinically employed intermittent injections of PTH (iPTH) to boost bone formation enhance IGF1 as well as TGFβ signaling pathways and thus increase osteoblastic glucose uptake and aerobic glycolysis [71,72,73].

### 3.2. Osteoblastic Effects on Overall Glucose Homeostasis

In the past few years, accumulating evidence of bone interference with overall glucose homeostasis has emerged. Osteoblasts secrete the hormone osteocalcin (OC), which is encoded by *BGLAP* [2]. In humans, the level of OC secretion depends on age and sex, as indicated by research carried out in the early nineties [74]. OC has various effects, depending on the state of carboxylation. When carboxylated, OC acts locally in the bone and increases the amount of calcium and hydroxyapatite stored, at least in vitro [75]. In diabetes, serum levels of OC increase during anti-diabetic treatment with good glycemic control [76]. Levels of uncarboxylated OC (uOC), which has multiple endocrine functions, are increased in a balanced diet with a caloric deficit of 500 kcal/day and correlate inversely with body fat [77,78]. Concerning glucose homeostasis, uOC increases pancreatic insulin secretion and adiponectin liberation from adipocytes, even though the clinical interactions of both are yet to be determined [79]. It is most likely released in an osteoclast-dependent manner, as glucose tolerance varies with osteoclast activity and uOC levels were increased up to 10-fold in mice with an OPG deletion [80]. In vivo experiments demonstrated that uOC-treatment prevents wild-type mice from developing diabetes when fed a high-fat diet [81]. Many in vitro studies identified G protein-coupled receptor family C member A (GPRC6A) as an uOC-receptor on pancreatic beta-cells, adipocytes and Leydig-cells of the testis [82,83,84]. In response to endogenous uOC stimulation, the release of adiponectin from adipocytes and pancreatic insulin secretion is mediated in both cases through activation of extracellular signal-regulated kinases (ERKs). While pancreatic insulin secretion involves synergistic activity of the Ras/Raf/MEK signaling pathway [85], adiponectin expression in adipocytes is enhanced through transcription factor PPARγ, which is activated through interaction with the cAMP responsive element, which also relates to B-Raf/MEK/ERK signaling [84]. Stimulation of GPRC6A via exogenous uOC admission leads to a synergistic increase in glucagon-like peptide-1 and subsequently decreased serum glucose levels in wild-type mice [86]. Reduced insulin secretion in response to uOC stimulation as well as overall decreased insulin levels have been observed in a conditional knockout of GPRC6A in pancreatic β-cells [87]. Inconsistently, mice with a global deletion of GPRC6A have been found to display normal bone and energy metabolism [88]. Details of the interaction and perhaps the involvement of different uOC receptors have not been confirmed yet and should be targeted in future research.

The uOC signaling pathway is of utmost interest, as it represents the only investigated effect of osteoblasts on glucose homeostasis. However, recent evidence indicates that other molecules apart from uOC might contribute to the interplay of bone and glucose [89]. In a murine model with an osteoblast-specific deletion of β-catenin, exogenous application of uOC failed to restore a normal energy metabolism, while treatment with OPG normalized all endocrine abnormalities [90]. In addition, a *Bglap* knockout strain without endocrine alterations has been reported [91,92]. Further research is needed to clarify whether an uOC supplementation could be of therapeutic interest or not.

## 4. Disruption of Osteoblast Function in Anorexia Nervosa and Diabetes

When global energy supply is nontransiently below average requirements, as seen in anorexia nervosia (AN), devastating disturbances of hormonal balance and bone loss occur. Most clinical studies found patients with AN to display lower levels of IGF1 and reduced serum levels of OC [5,93]. Inconsistently, in a separate case-control study, increased OC was measured in patients with AN [94]. These incoherent results may be caused through the inclusion of individuals with a binge eating/purging type in the study of Urano et al. [94], whereas Legroux-Gérot et al. [5] and Galusca et al. [93] only included individuals with the restricted form of AN in their studies. In vitro, depletion of glucose in the culture medium of human osteoblasts decreased ALPL activity and OC levels two-fold, whereas collagen expression was diminished by 20% [3]. Investigating the impact of energy depletion on bone turnover in mice, levels of OC in wildtype mice decreased with starvation, while only leptin-deficient mice displayed increased OC [95]. Furthermore, a shift from osteogenesis to adipogenesis in bone marrow was observed in an energy-deficiency model, similar to what is observed in patients with AN [96]. Overall, calorie restriction elicits multifactorial and complex processes locally and systemically and interferes with bone remodeling on multiple levels and requires further study. In summary, a tendency towards decreased osteoblastic markers under starvation, probably due to lack of substrates for energy generation, is found in literature. Increasing body weight to restore bone quality is therefore a crucial component of managing AN.

Diabetes is defined as a lack of insulin action with systemic consequences, including hyperglycemia, ketonuria and acidosis. The lack of insulin can be either due to an autoimmune dysfunction resulting in disruption of pancreatic beta-cells with a complete lack of insulin in diabetes mellitus type 1 (DM1) or due to insulin insensitivity in diabetes mellitus type 2 (DM2), most commonly in combination with the metabolic syndrome (obesity, dyslipidemia, hypertension and hyperglycemia). DM2 is diagnosed and monitored through measurement of serum HbA1c, which represents the fraction of glycolyzed hemoglobin and reflects blood sugar values of the last two to three months [97]. DM2 is widespread among populations of industrial countries, and its prevalence is increasing. In a matched cohort study, DM2 treatment with exogenous insulin surprisingly increases overall fracture risk up to 34% [98]. As diabetic patients display normal to high bone mineral density, the observed effects do not necessarily result from bone loss per se, but may as well be caused by hypoglycemic states with reduced alertness and increased risk of falling in addition to polyneuropathy. In accordance with that, HbA1c values lower than 6.5% were associated with a higher fracture risk in 650,000 male patients with diabetes [99].

Another crucial approach to understanding diabetes-induced bone fragility is given by the accumulating evidence on diminished bone quality in DM. Even though renal failure in advanced diabetic disease impairs hydroxylation of vitamin D into the active form calcitriol, osteoblast impairment most likely results from hyperglycemic metabolic states, which start to occur at earlier stages of the disease. Two independent clinical trials showed that osteoblastic ALPL secretion, OC levels and collagen synthesis are significantly lower in chronic hyperglycemia, and also after glucose-loading in healthy individuals [100,101]. In vitro and in vivo experiments found these effects to be accompanied by an impairment of osteoblastic Ca^2+^ uptake, as mineralized nodules display reduced calcium content, and femoral calcium levels are decreased by tendency in rodents with diabetes [102,103]. Nevertheless, human osteoblast cultures display varying responses towards a hyperglycemic environment in vitro. On the one hand, long-term incubation with a glucose concentration of 24 mM, as seen in DM, enhanced osteoblastic expression of OC and increased levels of RANKL, whereas levels of OPG decreased [104]. On the other hand, short-term incubation under hyperglycemia resulted in reduced ALPL, OC and collagen levels [3]. In rodent primary osteoblasts, hyperglycemic conditions led to increased proliferation but accounted for decreased calcium uptake as well, consistent with less matrix mineralization and diminished bone quality, respectively [105]. In in vitro studies with RUNX2 overexpression in osteoblasts, restored osteoblast differentiation and enhanced matrix mineralization were observed in a hyperglycemic environment [106]. Without exogenous manipulation, however, RUNX2 transcriptional activity is inhibited through post-transcriptional *O*-GlcNAcylation. Subsequently, osteoblastic differentiation and collagen synthesis are diminished under hyperglycemic conditions [107].

**Figure 3:**
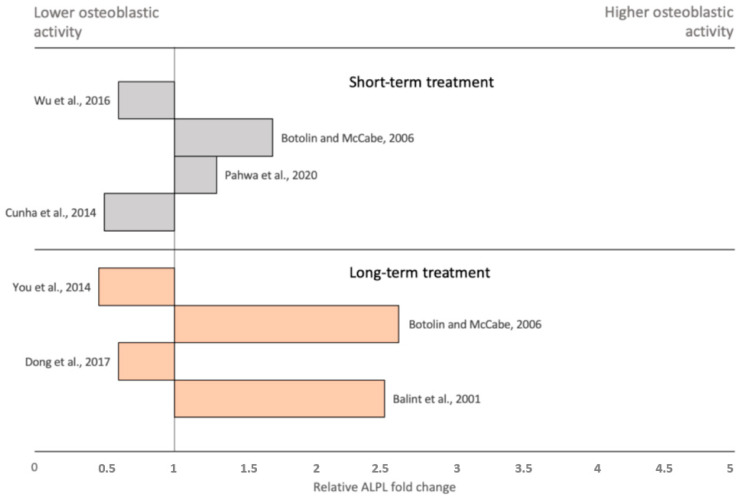
Bar graph displaying relative ALPL fold changes in MC3T3-E1 osteoblastic cells when incubated under hyperglycemic conditions (>15 mM Glucose) compared to normoglycemia (≙ 5.5 mM Glucose). Gray bars display the results of short-term (1–4 days) incubation; orange bars display the results of long-term (2–4 weeks) incubation. ALPL fold changes show, in summary, no significant differences, even though a tendency towards higher osteoblastic activity in chronic hyperglycemia can be observed in vitro.

Osteoblastic responses towards hyperglycemia are so far best researched in the immortal preosteoblastic cell line MC3T3-E1. Here, the expression of RANKL increased up to 10 fold and OPG up to 30 fold under incubation with high glucose levels, indicating a suppressive effect on osteoclasts and overall bone remodeling [4]. While Wu et al. [108] showed that short-term incubation with glucose levels higher than 15 mM increase ALPL mRNA expression, Pahwa et al. [109] observed decreased ALPL activity. In yet another study, short-term hyperglycemia resulted in higher levels of collagen type I but reduced the secretion of OC [110]. Likewise, the results of long-term incubations with high glucose concentrations vary. Decreased osteoblastic ALPL activity, OC secretion and collagen synthesis were observed upon exposure to 25.5 mM glucose chronically [111,112]. Inconsistently, increased ALPL activity under incubation with 30 mM glucose was observed in a different study [113]. Therefore, in vitro studies of osteoblastic gene expression within a hyperglycemic environment display complex findings with astonishing variations, even if parameters such as glucose concentration, start and duration of treatment and osteoblastic cell type are taken into consideration (Figure 3). Interestingly, long-term treatment displayed two outliers with highly increased osteoblastic ALPL activity in comparison to short-term treatment. This observation is consistent with the normal to high bone density observed clinically in individuals with DM2. Nevertheless, diabetes represents a complex process involving multiple organ systems, such as pancreatic beta cells, blood vessels, neurons and bone. Even if in vitro experiments are partially qualified for investigation of underlying molecular patterns, solid and reliable conclusions on clinically observed effects cannot be drawn, and future research employing laboratory animals as well as large-scale clinical trials will be indispensable for investigations of bone phenotype in diabetes.

On a molecular level, chronic hyperglycemia leads to an accumulation of advanced glycation end-products (AGEs). Subsequently, reactive oxygen species (ROS) are generated and reinforce oxidative stress [114]. AGEs and ROS are both toxic for osteoblast activity and have been identified to account for osteoblast disruption in diabetes in various trials [115]. In vitro studies showed that AGEs increase apoptosis of osteoblasts and prevent MSCs from differentiating into any kind of mature cell [116,117]. Further studies demonstrated an inhibition of osteoblast activity with reduced levels of ALPL and a reduced mineralization rate when incubated with AGE-modified molecules under normoglycemia, similarly to high-glucose concentrations [118,119]. In a different study, differentiation of murine stromal ST2 cells into osteoblasts was inhibited when incubated with AGEs, although hyperglycemic conditions alone had no impact on the process of differentiation [120]. Unfortunately, in all studies endogenous levels of AGEs in osteoblasts and osteoblast-like cells were not included, and values were given as “fold over control” compared to control group without treatment. This encourages the assumption that osteoblasts display AGEs even under physiological conditions, although this effect seems to take place at minor levels and therefore might be neglectable. To exert their effects, AGEs bind to the receptor for AGE (RAGE), which is expressed on MSCs and osteoblasts. Stimulation of RAGE has been found to inhibit the process of fracture healing in healthy and diabetic mice, as the area of regenerated bone decreased in either instance [121]. In vitro, osteoblastic AGE and RAGE expression increases under hyperglycemic conditions [122]. In vivo, comparison between diabetic rats and healthy controls showed a time-dependent increase of osteoblastic AGE, RAGE and ROS levels in flow cytometric analysis along with a decrease of bone volume and bone surface density in the diabetes group [123]. Evidence for higher bone resorption through induction of osteoblastic RANKL, and subsequently enhanced osteoclastogenesis was found as well [124].

In a different diabetic mice model, immunochemistry analysis of bone revealed higher oxidative damage alongside decreased bone formation rate and mineral density in diabetes-induced osteopenia [125]. Furthermore, diabetic mice displayed increased osteoblast apoptosis, which was found to be caused not only by AGE-dependent caspase activity, but also through ROS-dependent p53 activity, which results in the release of proapoptotic factor CytoC from damaged mitochondria into the cytosol [126]. Osteoblast markers, except for ALPL, were reduced, and a shift from osteogenesis towards adipogenesis was noticed in the presence of hyperglycemia-derived ROS in vitro [127]. Thus, pharmacologic approaches targeting ROS and AGEs might improve overall bone quality.

## 5. Implications for Anti-Diabetic Drugs

The first-line treatment for DM2, metformin, belongs to the class of biguanides and improves diabetes through inhibition of hepatic gluconeogenesis [128]. Further mechanisms, including decreased intestinal glucose absorption and increased tissue insulin-sensitivity, are still subject to research [129,130,131]. Positive effects on bone metabolism have been described in the literature as well. In vitro studies showed that metformin restores normal osteoblastic activity when cultured under hyperglycemic conditions and with AGEs, probably through inhibition of RAGE expression and induction of RUNX2 [132,133]. Comparable results have been observed in clinical trials, where metformin treatment reduced AGE levels and diminished ROS [134,135]. Metformin is also capable of restoring bone volume and stiffness in rodent models with estrogen-deficiency and energy-deficiency, respectively [136,137]. In a study with over 14,000 patients enrolled, osteoporosis and vertebral fractures are reduced up to 40% under treatment with metformin [138]. Metformin might contributes to improved fracture healing as well, as the rates of nonunion after femoral neck fractures are significantly lower in diabetic patients treated with metformin, even when compared to healthy subjects [139]. Overall, numerus osteoprotective characteristics of metformin were confirmed in vitro, in vivo and in clinical studies (for a detailed review, please see Bahrambeigi et al. [140]); however, further mechanistic research is required to decipher direct and indirect effects of metformin on bone cells. 

Incretines, such as glucagon-like peptide 1 (GLP-1), increase the release of insulin in response to higher blood glucose levels prostprandially and therefore represent another important target for anti-diabetic treatment. In experimental settings, GLP-1 receptor agonists have been found to increase osteoblastic differentiation of MSCs in a direct manner and to inhibit osteoclastogenesis via MAPK pathways [141,142]. Furthermore, increased trabecular volume as well as increased bone mineral density and mechanical strength was observed in various in vivo studies which employed diabetic and osteoporotic rodent models treated with GLP-1 receptor agonists exenatide and liraglutide [143,144,145]. Clinical data on GLP-1 receptor agonist treatment and bone phenotype is limited but has not shown any association with increased fracture risk heretofore [146]. A related group of anti-diabetics, dipeptidyl peptidase-4 (DPP4) inhibitors, were designed to prevent the degradation of GLP-1 [147]. In vitro, treatment with DPP4 inhibitors increased osteoblastic differentiation via upregulation of RUNX2, while no effects were observed when Wnt/β-catenin signaling was suppressed [148]. Moreover, treatment of rodents with DPP4 inhibitors increases trabecular bone mass and osteoblast activity in vivo, which has been suggested to be conveyed through direct and indirect mechanisms of action [149]. As IGF1 is another target substrate of DDP4 inhibitors, positive effects on bone quality might result from a decreased RANKL/OPG ratio [150]. While high serum cholesterol indirectly affects osteoblasts through oxidative damage and disruption of Wnt signaling [151], restored osteoblast function is observed when cholesterol reduced, for instance under treatment with DDP4 inhibitors. Results of a clinical meta-analysis indicated reduced fracture risk upon DPP4 inhibitor treatment [152]. In summary, DDP4 inhibitors mimic the pharmacologic effects of GLP-1 receptor agonists on bone turnover. Bone phenotypical changes secondary to anti-diabetic treatment with alpha glucosidase inhibitors, which augment levels of GLP-1 through distinct mechanisms, have not been investigated yet. Other recent anti-diabetic approaches include bile acid sequestrants, which lower glucose through activation of farnesoid X receptor (FXR). Even though a deletion of FXR decreased bone mineral density in mice [153], clinical data on bone phenotype changes are missing to date.

In contrast, other anti-diabetic drug classes have shown to have neutral effects on bone or even to diminish bone quality (Table 1). While sodium glucose co-transporter (SGLT2) inhibitors neither increase nor decrease fracture risk clinically, as reviewed by Donnan et al. [154], thiazolidinediones induce osteoclast differentiation via activation of transcription factor peroxisome proliferator-activated receptor gamma (PPARγ) and increase fracture risk up to 60% in a retrospective study [155,156]. The underlying molecular mechanisms of osteoclast activation include a PPARγ-dependent increase in expression of proto-oncogene c-Fos, which is located downstream of RANK and ultimately results in enhanced osteoclast differentiation [157]. Furthermore, the dopamine D2 agonist bromocriptine is used as anti-diabetic drug. Even though bromocriptine improved bone mineral density in patients with prolactinoma [158], these results should be evaluated carefully regarding the complex hormonal disturbances in pituitary disorders. In vitro, stimulation of dopamine signaling led to inhibited osteoclastogenisis, implying negative effects on bone quality [159]. Future research is required to address these findings in more detail and to adequately evaluate potential adverse or beneficial effects of anti-diabetic medications in patients with bone diseases. 

## 6. Conclusions

Comparatively intensive research has been carried out in the last few years to define the role of glucose metabolism in osteoblasts in health and disease. Even though not all contributing factors and regulatory mechanisms have been discovered yet, there is accumulating evidence that osteoblasts influence overall glucose homeostasis, and glucose itself represents the main nutrient for osteoblasts. Remarkably, energy production in osteoblasts is mainly mediated through the Warburg effect, which generates ATP from aerobic glycolysis and is also observed in tumor cells. Inconsistent findings regarding the hyperglycemic influence on osteoblasts remain a challenge, as clinically observed effects are only partially substantiated by in vitro studies. Nevertheless, different approaches towards improved osteoanabolic treatment have been found, and the repurposing of already established anti-diabetic drugs, including metformin, can improve bone quality in diabetic and perhaps also in healthy individuals. In this regard, further research on the interactions of glucose metabolism in osteoblasts with whole-body physiology could provide the basis for new therapeutic agents to improve bone quality and metabolic control.

## Figures and Tables

**Table 1:** Effects of antidiabetic drugs on bone: experimental and clinical observations.

Drug Class	Effect on Bone	Mechanism of Action	Model/Study Type	References
Biguanides (e.g., metformin)	‑ reduce osteoblastic cell death and hyperglycemia-derived AGE and ROS levels ‑ increase osteoblastic ALPL activity	‑ inhibition of caspase-3 activity, prevent overexpression of RAGE	in vitro	[130,131] reviewed by [139]
	‑ reduce serum AGE levels	-	clinical	[132,133]
	‑ restore bone stiffness and bone mineral density in estrogen-deficient and hypoglycemic rodent models	-	rat/mouse	[134,135]
	‑ reduce fracture risk in diabetic patients ‑ decrease rates of nonunion in diabetic individuals	-	clinical	[136,137]
GLP-1 receptor agonists	‑ induce MSC differentiation into osteoblasts, inhibit adipogenesis ‑ inhibit bone resorption through osteoclast suppression	‑ stimulate β-Catenin nuclear translocation through PKA/β-Catenin and PKA/PI3K/AKT/GSK3β signaling pathways ‑ inhibit NF-κB and MAPK signaling pathways	in vitro	[140,141]
	‑ restore bone volume ratio and bone mineral density in diabetes and estrogen-deficiency	-	rat/mouse	[142,143,144]
	‑ neutral effect on fracture risk	-	clinical	[145]
DPP4 inhibitors	‑ induce MSC differentiation into osteoblasts	‑ upregulation of RUNX2	in vitro	[147]
	‑ increase trabecular bone volume	‑ target IGF1, lower serum cholesterol hence oxidative damage and disruption of Wnt signaling	rat	[148]
	‑ tendency towards reduced fracture risk	-	meta-analysis	[151]
SGLT2 Inhibitors	‑ decrease in hip bone mineral density, increased OC serum levels accompanied by weight loss	-	clinical	[153]
	‑ neutral effects on fracture risk	-	clinical	[154,155]
	‑ partial preservation of trabecular bone volume in diabetes-induced bone disease	-	mouse	[156]
Thiazolidinediones	‑ increase fracture risk compared to untreated individuals and metformin users	-	clinical	[157]
	‑ increase osteoclastic cathepsin K expression ‑ inhibit osteoblast differentiation, no changes in ALPL activity	‑ PPARγ induced activation of c-Fos	in vitro	[158,159]
